# Safety and Clinical Outcome of Thrombolysis in Ischaemic Stroke Using a Perfusion CT Mismatch between 3 and 6 Hours

**DOI:** 10.1371/journal.pone.0025796

**Published:** 2011-10-10

**Authors:** Laszlo K. Sztriha, Dulka Manawadu, Jozef Jarosz, Jeff Keep, Lalit Kalra

**Affiliations:** 1 Department of Clinical Neuroscience, Institute of Psychiatry, King's College London, London, United Kingdom; 2 Department of Neuroradiology, King's College Hospital, London, United Kingdom; 3 General and Emergency Medicine, King's College Hospital, London, United Kingdom; Hôpital Robert Debré, France

## Abstract

**Objective:**

It may be possible to thrombolyse ischaemic stroke (IS) patients up to 6 h by using penumbral imaging. We investigated whether a perfusion CT (CTP) mismatch can help to select patients for thrombolysis up to 6 h.

**Methods:**

A cohort of 254 thrombolysed IS patients was studied. 174 (69%) were thrombolysed at 0–3 h by using non-contrast CT (NCCT), and 80 (31%) at 3–6 h (35 at 3–4.5 h and 45 at 4.5–6 h) by using CTP mismatch criteria. Symptomatic intracerebral haemorrhage (SICH), the mortality and the modified Rankin Score (mRS) were assessed at 3 months. Independent determinants of outcome in patients thrombolysed between 3 and 6 h were identified.

**Results:**

The baseline characteristics were comparable in the two groups. There were no differences in SICH (3% v 4%, p = 0.71), any ICH (7% v 9%, p = 0.61), or mortality (16% v 9%, p = 0.15) or mRS 0–2 at 3 months (55% v 54%, p = 0.96) between patients thrombolysed at 0–3 h (NCCT only) or at 3–6 h (CTP mismatch). There were no significant differences in outcome between patients thrombolysed at 3–4.5 h or 4.5–6 h. The NIHSS score was the only independent determinant of a mRS of 0–2 at 3 months (OR 0.89, 95% CI 0.82–0.97, p = 0.007) in patients treated using CTP mismatch criteria beyond 3 h.

**Conclusions:**

The use of a CTP mismatch model may help to guide thrombolysis decisions up to 6 h after IS onset.

## Introduction

Intravenous thrombolytic treatment for ischaemic stroke (IS) using a recombinant tissue plasminogen activator (tPA) within 3 h of symptom onset has been well established by randomised controlled studies [1], the results of which have been replicated in observational registries [2]. The European Cooperative Acute Stroke Study (ECASS) III study has demonstrated that tPA is more efficacious than placebo in the 3–4.5-h window [3]. Results from the updated SITS-ISTR registry of thrombolysed patients support the benefit of treatment in the 3–4.5-h window, although with odds lower for a good functional state and higher for symptomatic intracerebral haemorrhage (SICH) and mortality as compared with treatment within 0–3 h [4]. A recent pooled analysis of randomised controlled thrombolysis trials with patients selected by clinical symptoms and non-contrast CT (NCCT) within 0–6 h found that the odds of a favourable outcome decreased and those of mortality rose with increase of the onset-to-treatment time (OTT), and concluded that patients benefit from tPA when treated up to 4.5 h [1]. However, within a large cohort of patients there might be some who still derive benefit from treatment up to 6 h [1].

Penumbral imaging may be an approach with which to select patients likely to benefit from thrombolysis in an extended time window [5]. Results from large observational studies suggest that multimodal MRI-guided thrombolysis between 3 and 6 h has safety and efficacy outcomes comparable to those of NCCT-based thrombolysis within 0–3 h [6]. A target MR mismatch profile associated with the best outcomes has also been suggested [7]. The use of MRI for patient selection in acute stroke, however, is frequently limited, reasons including restricted access to hyperacute MRI in many hospitals, and established contraindications such as certain implanted devices, or uncooperative or claustrophobic individuals. Perfusion CT (CTP) has much fewer constraints, and is available on many scanners currently in use. Interestingly, there have been no published reports of studies in which patients were selected exclusively with CTP to undergo intravenous tPA therapy in an extended time window [8].

The hypothesis of this study was that a structured assessment of CTP in the clinical routine can identify patients with a target mismatch profile who are likely to benefit from late thrombolysis. The objective was to compare safety and efficacy outcomes of intravenous thrombolysis in patients presenting at 3–6 h, assessed with a simple but structured evaluation of CTP by using the semiquantitative Alberta Stroke Program Early CT Score (ASPECTS) scale [9], with those presenting within 0–3 h, assessed with NCCT.

## Methods

### Ethics Statement

All patients in this study received treatment according to standard clinical practice. The routine clinical use of tPA beyond current licence in selected patients was approved by the King's College Hospital Novel Procedures Committee. All participants or their surrogates provided informed verbal consent, which was documented in the medical notes as per the institutional Clinical Governance procedures. The analysis of data collected during the routine clinical management of patients was in compliance with the institutional Clinical Governance guidelines to monitor the safety of thrombolysis.

### Patients

Data were prospectively collected and entered into a database on all consecutive patients with anterior circulation IS who were thrombolysed according to a standard clinical protocol within 6 h of symptom onset at a single stroke centre between 2007 and 2009. Baseline patient characteristics and OTT and arrival-to-treatment time (ATT) were recorded. The National Institute of Health Stroke Scale (NIHSS) score was documented by certified assessors at baseline and at 24 h after thrombolysis [10]. The modified Rankin Score (mRS) was recorded to characterize the level of functioning both pre-morbidly and at 3 months post-thrombolysis [11]. All analyses were done retrospectively on the prospectively collected dataset.

### Imaging

CT examinations were performed on a 16-slice multidetector scanner (LightSpeed, GE Healthcare, USA). All patients underwent routine NCCT to exclude haemorrhage and to assess early ischaemic changes. The negative ordinal ASPECTS scale (range 10-0) was used to evaluate the extent of ischaemic change within the middle cerebral artery (MCA) territory [9]. CTP was undertaken immediately following NCCT in IS patients who presented between 3 and 6 h. Image acquisition was performed as a 50-sec cine series beginning 5 sec after a power injection (Medrad Power Injector, Medrad, USA) of 50 ml of a non-ionic iodinated contrast (Ominpaque 300 mg/mL, GE Healthcare, UK) at 4 ml/s through a 18–20 gauge antecubital intravenous catheter. The imaging parameters were 80 kVp, 200 mAs, and a rotation time of 1 sec. Coverage consisted of two contiguous slices of 10-mm thickness positioned parallel and superior to the orbital roof, with the more caudal section at the level of the basal ganglia and internal capsule. The dynamic perfusion CT source images were analysed by using a semi-automated postprocessing software (CT Perfusion 2.6.9, GE Medical Systems, USA) that generated colour maps of cerebral blood volume (CBV), cerebral blood flow (CBF) and mean transit time (MTT). The colour scales were set at 0–10 ml/100 g for the CBV, 0–100 ml/100 g/s for the CBF, and 0–15 s for the MTT maps. The arterial and venous inputs were obtained from the anterior cerebral artery and the superior sagittal sinus, respectively. A structured method of visual assessment, applying the semiquantitative ASPECTS technique, was used to read the CTP maps. Areas showing normal CBV and MTT were scored as 1 and those demonstrating an impairment as compared to the contralateral hemisphere were rated as 0. ASPECTS mismatch was defined as the CBV ASPECTS minus the MTT ASPECTS. The ASPECTS mismatch is therefore a positive ordinal scale ranging from 0 to 10. Images were read by two trained assessors not involved in the acute care of the patients.

Follow-up NCCT was performed routinely at 24 h, or at any time when a neurological deterioration occurred. The presence of any intracerebral haemorrhage on follow-up imaging was recorded.

### Interventions

In accordance with the routine in-house clinical protocol, IS patients presenting within 3 h of stroke onset who had an ASPECTS ≥7 on NCCT and no contraindications to tPA were thrombolysed. IS patients presenting between 3 and 6 h post onset were eligible for thrombolysis if they had no contraindications to tPA (apart from time), and demonstrated a target mismatch profile defined as a CBV ASPECTS ≥7 and an ASPECTS mismatch ≥2. Intravenous tPA (0.9 mg/kg, maximum 90 mg) was given as an infusion over 60 min, with 10% of the total dose administered as a bolus.

### Analyses

Efficacy end-points included disability at 3 months, as assessed by means of the mRS, with scores 0–2 indicating functional independence, and 0–1 referring to minimal or no disability. Another efficacy end-point was the change in the NIHSS score at 24 h. Safety end-points included overall mortality at 3 months, any intracerebral haemorrhage (ICH), and SICH as defined according to the SITS-MOST criteria [2].

Baseline characteristics, clinical outcome and ICHs were compared between patients thrombolysed in the 0–3-h window using NCCT and those treated at 3–6 h using CTP mismatch criteria. Following the publication of the ECASS III trial [3], groups of patients thrombolysed using CTP criteria in the 3–4.5-h and 4.5–6-h windows were also compared. Comparisons were undertaken with the t-test, Mann-Whitney U-test, chi-square or Fisher exact test as appropriate. Odds ratios (OR) with 95% confidence intervals (CI) were calculated for the outcome measures. ORs adjusted for differences in baseline characteristics, using logistic regression, are also reported. Stepwise logistic regression modelling was performed to identify factors associated with mRS 0–2 at 3 months in patients thrombolysed in the 3–6-h window. Independent variables included age, sex, baseline NIHSS score, blood pressure and OTT. The interactions between age×OTT, NIHSS score×OTT and age×NIHSS score were also examined. Inter-rater reliability was evaluated by using the intraclass correlation coefficient derived from a 2-way random effects model for absolute agreement. Two-sided p values are reported, with the level of significance set at <0.05. SPSS version 17.0 (SPSS Inc., USA) was used for analyses.

## Results

Three hundred and nineteen thrombolysed IS patients with a known time of onset and symptoms/signs attributable to MCA involvement were entered into our database between January 2007 and December 2009. We excluded 24 patients in the 3–4.5-h window who were thrombolysed without assessment of a CTP mismatch, after the results of the ECASS III study had been published [3]. We also excluded 41 patients in the 0–3-h time window in whom CTP was performed with the purpose of establishing a vascular cause of their symptoms (Figure S1). The final analysis included 254 patients, of whom 174 (69%) were thrombolysed at 0–3 h using NCCT, and 80 (31%) at 3–6 h using CTP mismatch criteria. Of those thrombolysed at 3–6 h, 35 (44%) were within 3–4.5 h and 45 (56%) between 4.5 and 6 h.

Baseline patient characteristics are compared in Table 1. The median age was 74 years, and approximately half were male in both groups. Atrial fibrillation was more common among those treated at 0–3 h (43% v 30%, p = 0.045). There was no significant difference in the baseline NIHSS (14 v 13, p = 0.30). The median ATT was 10 minutes longer in the 3–6-h group assessed with CTP, a significant difference in comparison with the 0–3-h NCCT group.

**Table 1:** Baseline characteristics and treatment times.

	0–3 h NCCT (n = 174)	3–6 h CTP mismatch (n = 80)	p value
Median age (year)	74 (65–82)	74 (62–81)	0.60
Male	92 (53%)	43 (54%)	0.90
Hypertension	118/173 (68%)	48 (60%)	0.20
Diabetes	35/173 (20%)	10 (13%)	0.14
Hypercholesterolaemia	55/167 (33%)	28/77 (36%)	0.60
Current smoker	33/154 (21%)	19/74 (26%)	0.47
Atrial fibrillation	74/171 (43%)	24 (30%)	0.045
Mean systolic BP (mmHg)	146 (126–166)	144 (122–166)	0.56
Mean diastolic BP (mmHg)	80 (66–94)	80 (66–94)	0.94
Mean baseline blood glucose (mmol/l)	7.1 (4.4–9.8)	6.8 (4.9–8.7)	0.43
Median baseline NIHSS	14 (9–19)	13 (7–18)	0.30
Median OTT (min)	120 (95–145)	280 (240–315)	<0.001
Median ATT (min)	60 (35–85)	70 (50–118)	<0.001

Data are median (interquartile range), number (%), or mean (±1 standard deviation).

BP, blood pressure at baseline.

**Figure 1:**
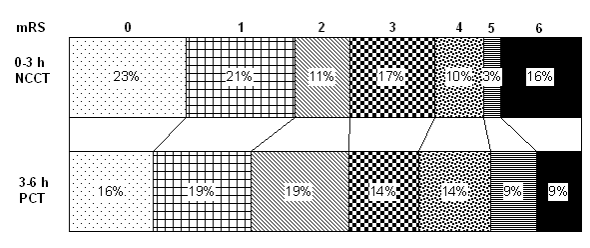
Distribution of scores on the mRS.

The rate of SICH was 3% in patients thrombolysed at 0–3 h using NCCT, and 4% (p = 0.61) among those in the 3–6 h window receiving CTP (Table 2). There were no differences in ICH, fatal ICH or mortality. The change in the median NIHSS score at 24 h after thrombolysis (−5 v −5, p = 0.55) and the proportion of patients with a favourable functional outcome (mRS 0–2) at 3 months were comparable in the two groups (55% v 54%, p = 0.96) (Table 2, Figure 1). The OR for every outcome remained statistically non-significant after adjustment for atrial fibrillation and ATT. Comparisons between patients thrombolysed at 3–4.5 h and those treated at 4.5–6 h revealed that more older women were treated in the 4.5–6-h window, but there were no differences in ICHs, mortality or other clinical outcomes (Table 3).

**Table 2:** Comparison of outcome measures.

	0–3 h NCCT (n = 174)	3–6 h CTP mismatch (n = 80)	OR [95% CI]	p value	Adjusted OR [95% CI]	p value
Haemorrhage						
Any ICH	12/171 (7%)	7/79 (9%)	1.29 [0.49–3.41]	0.61	1.86 [0.67–5.16]	0.24
SICH	5/171 (3%)	3/79 (4%)	1.31 [0.31–5.63]	0.71	3.0 [0.59–15.21]	0.19
Fatal ICH	4/171 (2%)	0/79		0.31		
Neurological function						
Median 24 h NIHSS	7 (2–13)	6 (2–13)		0.93		
Median change in NIHSS from baseline	−5 (−9 to −2)	−5 (−8 to −2)		0.55		
Functional level at 3 months						
mRS 0–1	69/157 (44%)	28/79 (35%)	0.70 [0.40–1.22]	0.21	0.63 [0.35–1.15]	0.14
mRS 0–2	86/157 (55%)	43/79 (54%)	0.99 [0.57–1.70]	0.96	0.93 [0.52–1.66]	0.81
mRS 6 (death)	25/160 (16%)	7/79 (9%)	0.53 [0.22–1.27]	0.15	0.77 [0.31–1.95]	0.58

Values in parentheses for Neurological function indicate interquartile range.

**Table 3:** Comparison of patients treated in the 3–4.5-h and 4.5–6-h windows using CTP mismatch.

	3–4.5 h (n = 35)	4.5–6 h (n = 45)	p value
**Baseline characteristics**			
Median age (year)	69 (51–78)	74 (65–80)	0.023
Male	24 (69%)	19 (42%)	0.019
Hypertension	17 (49%)	31 (69%)	0.066
Diabetes mellitus	5 (14%)	5 (11%)	0.74
Hypercholesterolaemia	11/32 (34%)	17 (38%)	0.76
Current smoker	12/33 (36%)	7/41 (17%)	0.059
Atrial fibrillation	8 (23%)	16 (36%)	0.22
Mean systolic BP (mm Hg)	144 (119–169)	145 (127–163)	0.81
Mean diastolic BP (mm Hg)	79 (64–94)	81 (68–94)	0.61
Mean baseline blood glucose (mmol/l)	6.7 (4.5–8.9)	6.8 (5.0–8.6)	0.99
Median baseline NIHSS	11 (7–18)	13 (8–18)	0.38
Median OTT (min)	230 (197–243)	310 (285–330)	<0.001
**Outcome measures**			
Any ICH	2/34 (6%)	5 (11%)	0.69
SICH	1/34 (3%)	2 (4%)	1.00
Fatal ICH	0/34 (0%)	0 (0%)	
Median 24-h NIHSS	4 (2–10)	7 (3–13)	0.39
Median change in NIHSS from baseline	−5 (−8 to −2)	−5 (−8 to −1)	0.91
mRS 0–1 at 3 months	12/34 (35%)	16 (36%)	0.98
mRS 0–2 at 3 months	20/34 (59%)	23 (51%)	0.50
mRS 6 (death) at 3 months	5/34 (15%)	2 (4%)	0.13

Data are median (interquartile range), number (%), or mean (±1 standard deviation).

BP, blood pressure at baseline.

A low baseline NIHSS score was the only independent determinant of a favourable functional outcome (mRS 0–2) (OR 0.89, 95% CI 0.82–0.97, p = 0.007) in patients treated using CTP mismatch criteria beyond 3 h. There were no significant interactions between NIHSS score, OTT or age.

The interobserver reliability scores for CBV, MTT and mismatch ASPECTS were 0.82, 0.79 and 0.81, respectively (p<0.0001 for each).

## Discussion

This study demonstrates that SICH and the functional outcome following thrombolysis of IS patients in the 3–6 h window using a structured assessment of CTP mismatch are comparable to those for patients thrombolysed at 0–3 h using NCCT alone. Within the 3–6-h group, there were no differences in SICH or functional outcome between patients thrombolysed at 3–4.5 h and those treated at 4.5–6 h, though the numbers in each group were relatively small. The performance of CTP on average added 10 minutes to the ATT; however, this may not be clinically disadvantageous as the OTT did not prove to be a significant determinant of outcome among our patients exhibiting a CTP mismatch in the 3–6-h window, a finding similarly observed in studies using multimodal MR for patient selection [6], [12]. Our outcomes of late thrombolysis using CTP mismatch were comparable to those of previous studies using MR mismatch criteria [6], [12].

There are some considerations concerning the use of CTP to guide thrombolysis decisions in clinical settings. Image analysis using thresholds for different CTP parameters and a quantitative determination of mismatch volume may involve complicated calculations frequently requiring additional software, which can easily lead to significant delays in treatment. In contrast, the semiquantitative ASPECTS methodology, originally designed for the assessment of early ischaemic changes in the MCA territory on NCCT, is a quick tool that has been applied to CTP too [13]. ASPECTS on CBV maps has been found to be a more accurate predictor of irreversibly injured tissue and clinical outcome than NCCT [13], with a good correlation to the DWI lesion on MRI [14]. We believe that the CBV ASPECT threshold of ≥7 in our study identified patients with a relatively small infarct core that is associated with a more favourable prognosis [13]. The definition of ASPECT mismatch was initially introduced in multimodal MRI to facilitate mismatch assessment in a simple and reliable fashion, without the need for time-intensive calculations [15]. The CBV and MTT maps of CTP seem to provide a good estimate of the extent of tissue at risk [13], which can also be expressed as an ASPECT mismatch score. A CTP ASPECT mismatch score of ≥2, as used in our penumbral model for patient selection, has been found to be the optimum cut-off point for a volumetric mismatch of ≥100% [16]. The previously reported excellent interrater reliability for ASPECT scoring of CTP maps was likewise confirmed by our study [13]. We therefore conclude that a rapid structured assessment of mismatch on CTP maps has the potential to reliably guide thrombolytic treatment in IS patients in an extended time window. However, we are aware of concerns regarding the CTP technique itself, inclusive of limited brain coverage, the lack of clearly defined thresholds for salvageable tissue, and the variability of results depending on the type of postprocessing software, highlighting the need for validation and standardization of CTP methods [8], [17].

There are some limitations to this study. Although the case numbers in this single centre were obviously lower than in multicentre registries, they compare very well with those in studies of similar design looking at multimodal MRI-guided thrombolysis in an extended time window [12]. A potential further limitation is the lack of a control group of patients without a mismatch profile. Results from the Diffusion and Perfusion Imaging Evaluation for Understanding Stroke Evolution (DEFUSE) study, however, suggest that early reperfusion is not beneficial in patients with a ‘no mismatch’ profile [7]. Moreover, there were no non-thrombolysed controls with a mismatch profile, and we are unaware of a single placebo-controlled study of intravenous tPA treatment of patients exhibiting a mismatch on CTP. As the results of the ECASS III trial became available only towards the end of our study period [3], it was not a pre-defined objective of this investigation to include patients treated by using only a NCCT in the 3–4.5-h window. Since this was an open study, outcomes may have been assessed more favourably in the 3–6-h group. Although this could potentially have affected NIHSS and mRS assessments, neither mortality nor the detection of a haemorrhage on CT were subject to this bias. The results of this study can be generalized to patients with a stroke in the anterior circulation. Those with symptoms in the posterior circulation, a territory where CTP is known to underperform, were excluded.

A recent meta-analysis of mismatch-based thrombolysis after 3 h of stroke onset, including trials of desmoteplase in a significant proportion, did not demonstrate an improvement in clinical outcome, despite increased reperfusion [18]. This is in contrast with the conclusions of multimodal MRI-based cohort studies of tPA and also of our own investigation [6], [12]. The ongoing phase III randomised controlled Extending the Time for Thrombolysis in Emergency Neurological Deficits (EXTEND) study is expected to explore the benefit of multimodal MRI-based tPA treatment in the 3–9-h window [19], and we propose that similar trials using CTP mismatch criteria are also warranted, with a potential to nearly double the number of IS patients receiving thrombolysis [20].

## Supporting Information

Figure S1 Flowchart of patient inclusion.
(PDF)
