# Supplementary figures and images for: Safety and Clinical Outcome of Thrombolysis in Ischaemic Stroke Using a Perfusion CT Mismatch between 3 and 6 Hours
Source: PLoS One. 2011 Oct 10;6(10):e25796. doi: 10.1371/journal.pone.0025796 (PMC3189921; doi:10.1371/journal.pone.0025796)

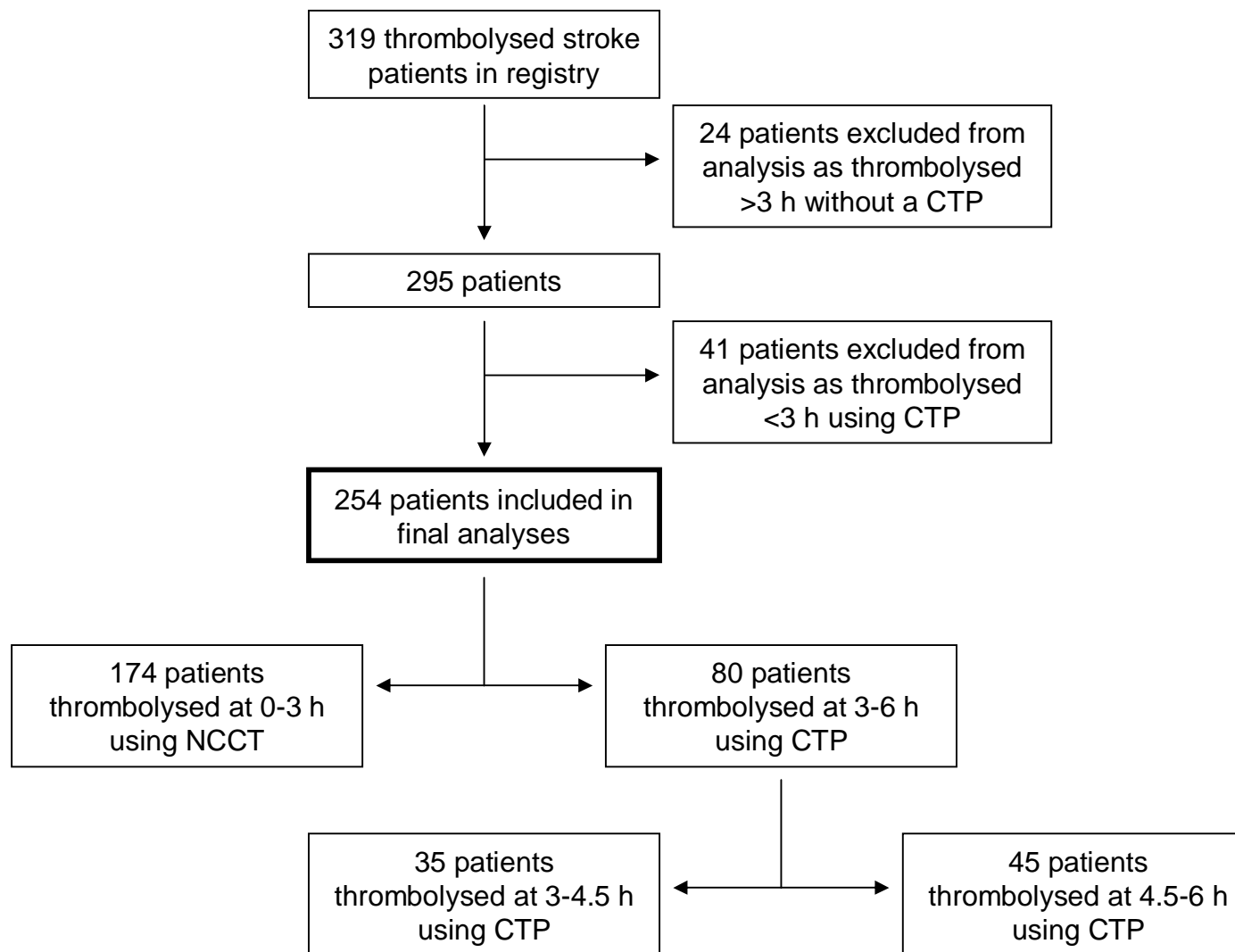

Supplement: Figure S1 — Flowchart of patient inclusion. (PDF) [file pone.0025796.s001.pdf]
